# Transient decrease in serum potassium level during ischemic attack of acute coronary syndrome: Paradoxical contribution of plasma glucose level and glycohemoglobin

**DOI:** 10.1186/1475-2840-12-4

**Published:** 2013-01-04

**Authors:** Hiroshi Sekiyama, Tomohisa Nagoshi, Kimiaki Komukai, Masato Matsushima, Daisuke Katoh, Kazuo Ogawa, Kosuke Minai, Takayuki Ogawa, Michihiro Yoshimura

**Affiliations:** 1 Division of Cardiology, Department of Internal Medicine, The Jikei University School of Medicine, 3-25-8, Nishi-Shinbashi, Minato-ku, Tokyo, 105-8461, Japan; 2 Division of Clinical Epidemiology, The Jikei University School of Medicine, 3-25-8, Nishi-Shinbashi, Minato-ku, Tokyo, 105-8461, Japan

**Keywords:** Potassium level, Acute coronary syndrome, Glucose level, Diabetes

## Abstract

**Background:**

Although a decrease in serum potassium level has been suggested to be a fairly common observation in acute coronary syndrome (ACS), there have so far been no definitive reports directly demonstrating the transient potassium decrease (the potassium dip) during ischemic attack of ACS compared to stable phase in individual patients. To understand the pathophysiological significance of the potassium dip, we examined the changes in serum potassium level throughout ischemic attack and evaluated the clinical factors affecting it.

**Methods:**

The degree of the potassium dip during ischemic attack (as indicated by ΔK, ΔK = K at discharge − K on admission) was examined in 311 consecutive patients with ACS who required urgent hospitalization in our institution.

**Results:**

Serum potassium level during ischemic attack was significantly decreased compared to that during stable phase (P < 0.001). Multiple regression analysis revealed that plasma glucose level during attack was the sole factor which was positively correlated with ΔK (P < 0.01), while HbA1c level was negatively correlated (P < 0.05). The medication profiles and renal function had no impact on ΔK. A longer hospitalization period, higher incidence of myocardial infarction and higher peak creatine kinase level were observed in patients with a larger ΔK.

**Conclusions:**

We have clearly demonstrated that there is a transient decrease in serum potassium level during ischemic attack of ACS compared to stable phase. The degree of the potassium dip was tightly correlated with glucose level, which overwhelmed the diabetic condition, and it also indicates the disease severity. The present study therefore promotes awareness of the significance of monitoring potassium level in parallel with glucose level in patients with ACS.

## Background

A decrease in serum potassium (K) level has been suggested to be a fairly common observation in patients with acute coronary syndrome (ACS) [1-4], which has been shown to increase the risk of cardiac events, including lethal ventricular arrhythmias [5-7]. In addition, a decrease in K level generally induces vasoconstriction [8], which leads to further ischemia, thereby producing a vicious cycle. The optimal range of K level in ACS has been recently discussed and reviewed [9], and the importance of potassium homeostasis during ischemic attack was thus clarified. However, little is known about the pathophysiological significance of potassium kinetics during ACS attack. Moreover, there have so far been few studies directly demonstrating the transient relative decrease in K level during ischemic attack compared to stable phase in individual patients (rather than its absolute value on admission). In fact, the mean value of K concentration on admission was around 4.0 mmol/L in all of the previous reports, which is not technically defined as hypokalemia. To see this transient K decrease, “the potassium dip,” during ischemic attack, it would be necessary to evaluate the fluctuation of K level in individuals by comparing the data during an attack to those during stable phase. We hypothesized that the degree of the decrease in serum K level may indicate the disease severity of ACS. To understand the pathophysiological significance of the potassium dip in ACS and to ensure that patients have an optimal serum K level during the acute phase of ischemic attack, we herein examined the changes in K level throughout ischemic attack and evaluated the clinical factors affecting it.

## Methods

### Study patients

The study protocol was approved by the ethics committee of The Jikei University School of Medicine (21-027(5605)).

Patients with ACS who required emergency admission to The Jikei University Hospital from January 2006 to December 2011 were included in this study. ACS was defined as the presence of myocardial infarction (MI) or unstable angina pectoris, as described previously [10]. Briefly, the diagnosis of MI required the presence of any two of the following three criteria: (1) a history of cardiac chest pain lasting at least 30 minutes; (2) typical electrocardiographic changes (i.e. ≥ 0.1 mV ST elevation in at least one standard lead or two precordial leads, ≥ 0.1 mV ST depression in at least two leads, abnormal Q waves, or T-wave inversions in at least two leads); (3) an increase in serum creatine kinase (CK) level to more than twice the upper limit of the normal range. All patients with MI were admitted to the hospital within 1 week of the onset. Unstable angina pectoris was diagnosed when patients fulfilled the criteria for the Braunwald clinical classification without an increase in serum CK level [11]. Patients were excluded if they were receiving or beginning to receive dialysis, were taking potassium controlling agents, or died from any cause during hospitalization. Based on these selection criteria, 311 consecutive patients, including 188 with MI, were enrolled.

### Data collection

The baseline characteristics, including the clinical parameters and the biochemical data, were collected retrospectively from the hospital medical records. The serum K level on admission was defined as K during ischemic attack, and the serum K level at the time of discharge was defined as K during stable phase. The degree of the potassium dip during ischemic attack (as indicated by ΔK) was calculated from the difference between K at discharge and K on admission:

(1) ΔK=Katdischarge-Konadmission

In other words, a larger ΔK indicates a greater decrease in serum K level during ischemic attack in comparison to K level during the stable phase.

The serum K level before admission was available in 85 patients that had incidentally undergone blood tests within six months before the ischemic attack. All other biochemical data, including plasma glucose level, were measured at the time of admission, except for peak CK level. Diabetes mellitus (DM), hypertension, and dyslipidemia were defined as described previously [10,12]. The estimated glomerular filtration rate (eGFR) was calculated as described previously [12]. The hemodynamic parameters, including left ventricular ejection fraction (LVEF), were measured on the day of admission.

### Definitions of the medication profiles

To evaluate the involvement of renin-angiotensin-aldosterone system inhibitors (RAAS-I) and diuretics, we examined the influence of the changes in each medication profile as follows: “no change” indicates that those medications were or were not taken both on admission and at the time of discharge; “newly administered” indicates that those medications were not taken on admission but were introduced during hospitalization; “discontinuation” indicates that those medications were taken on admission but were discontinued during hospitalization.

### Statistical analysis

Continuous variables were expressed as the means ± SD. To compare the serum K level between groups, the statistical analyses were performed using one way repeated measure analysis of variance, followed by a Bonferroni multiple comparison correction for three phases and paired sample *t*-test for two groups. The statistical analyses were performed using one way analysis of variance (ANOVA) followed by Scheffe’s test to assess the influence of β-blocker use on admission and the changes in the medication profiles of RAAS-I and diuretics on ΔK. To assess the determinants of ΔK, multiple regression analyses were performed after simple regression analyses were performed. The patients’ age, blood pressure, B-type natriuretic peptide (BNP), body mass index (BMI), glycohemoglobin (HbA1c), eGFR, glucose, K level on admission, LVEF, change in medication profile of RAAS–I and diuretics, and use of β-blockers on admission were included as variables. In the multiple regression analysis, indicator variables were employed as follows; one indicator variable coded as 0/1 for variable with two categories (use of β-blockers on admission) and two indicator variables for variables with three categories (changes in medication profiles of RAAS-I and diuretics) were generated. The regression coefficient of each indicator variable indicates the effect of that category in comparison to “no change” in each medication profile (as a basic category). All patients were divided into two groups based on the median value of ΔK and serum K on admission, to investigate the association of ΔK and serum K on admission with the disease severity and clinical course. Continuous variables were evaluated by the Welch test for unequal variances, Student’s *t*-test for equal variances and the Chi-square test for categorical variables. P < 0.05 was considered to be statistically significant. All data were statistically analyzed using the SPSS software package, version 11.5 (SPSS Inc., Chicago, IL).

## Results

**Figure 1:**
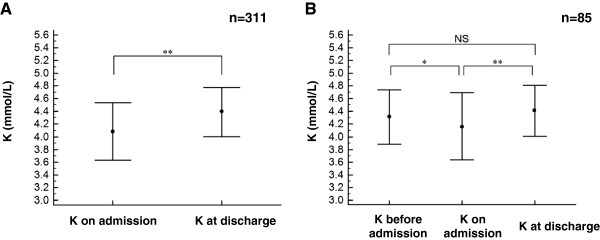
**The time course of the serum potassium concentration (K) profile. A**. The average ± SD of K level in the total study patients (n = 311) on admission (during ischemic attack) and at discharge (during stable phase) (paired *t*-test, ^**^P < 0.001). **B**. The K level in the 85 patients for whom the data before admission were available (^**^P < 0.001 and ^*^P < 0.025 by one way repeated measure analysis of variance, followed by a Bonferroni multiple comparison correction for three phases). NS; not significant.

The baseline clinical characteristics of the 311 patients are shown in Table 1. The mean serum K level was 4.1 ± 0.4 mmol/L during ischemic attack (on admission) and 4.4 ± 0.4 mmol/L during stable phase (at discharge). K on admission was significantly decreased compared to K at discharge (Figure 1A, P < 0.001) and K before admission (Figure 1B, P < 0.025). The mean plasma glucose level on admission was 155 ± 68 mg/dL and the mean HbA1c was 6.0 ± 1.2% (Table 1). A total of 60.5% of the patients were diagnosed with MI and 39.2% were diagnosed with type 2 DM.

**Table 1:** Baseline characteristics (n = 311)

Age, years	63 ± 12
Male, gender (%)	260 (83.6)
Height, cm	167 ± 34.2
Weight, kg	66.4 ± 12.5
BMI, kg/m2	24.1 ± 3.9
BP, mmHg	
Systolic	137 ± 27
Diastolic	78 ± 17
Mean	98 ± 19
K on admission, mmol/L	4.1 ± 0.4
K at discharge, mmol/L	4.4 ± 0.4
eGFR, mL/min/1.73m2	71.1 ± 22.6
Cr, mg/dL	0.9 ± 0.3
HbA1c, %	6.0 ± 1.2
Glucose, mg/dL	155 ± 68
BNP, pg/mL	144 ± 317
LVEF, %	54 ± 10.9
Time of hospital stay, days	12.3 ± 9.9
Myocardial infarction (%)	188 (60.5)
Unstable angina (%)	123 (39.5)
Diabetes mellitus (%)	122 (39.2)
Hypertension (%)	212 (68.2)

Of the 311 patients, 125 (40.2%) had taken one or more RAAS-I and/or diuretics on admission and 260 (83.6%) had taken these agents at the time of discharge (Additional file 1). Forty-nine patients (15.6%) had taken β-blockers on admission (β1 selective β-blockers: 26 patients; non-selective β-blocker: 23 patients) and 131 (42.1%, β1 selective β-blockers: 21; non-selective β-blocker: 110) had taken β-blockers at the time of discharge.

**Figure 2:**
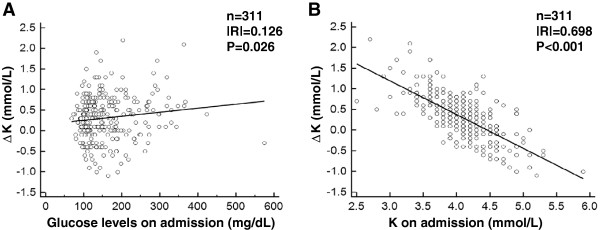
**The results of the simple regression analyses.** The simple regression analyses between plasma glucose level on admission (during ischemic attack) and ΔK (**A**), between serum K level on admission (during ischemic attack) and ΔK (**B**) are shown. ΔK = K at discharge - K on admission.

We performed a simple regression analysis to evaluate the determinants of ΔK (Table 2). Plasma glucose level during ischemic attack (on admission) showed a significantly positive correlation with ΔK (Figure 2A, P = 0.026). On the other hand, there was a negative correlation between ΔK and K on admission (Figure 2B, P < 0.001). ΔK was not associated with HbA1c and eGFR on admission.

**Table 2:** The results of a simple regression analysis of ΔK (n = 311)

**Explanatory variables**	**Regression coefficients**	**Standard error**	**Standard regression coefficients**	**F**	**P**
K on admission	−0.815	0.048	−0.698	294.07	<0.001
Glucose on admission	0.001	0.0004	0.126	5.026	0.026
HbA1c	−0.020	0.024	−0.048	0.7	0.404
eGFR	−0.001	0.001	−0.063	1.213	0.272
BNP	−0.0002	0.0001	−0.101	3.181	0.075
LVEF	−0.005	0.003	−0.095	2.808	0.095
BMI	0.010	0.008	0.075	1.726	0.19
Age	−0.002	0.002	−0.037	0.414	0.52
Blood pressure (mean)	0.002	0.002	0.082	2.084	0.15

ΔK = K at discharge − K on admission.

K: potassium, eGFR: estimated glomerular filtration rate, BNP: B-type natriuretic peptide, LVEF: left ventricular ejection fraction, BMI: body mass index, BP: blood pressure.

**Figure 3:**
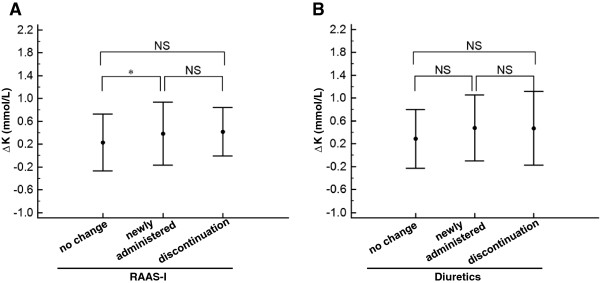
**The comparison of ΔK among the changes in the medication profiles.** The comparison of ΔK among indicated changes in the profiles of rennin-angiotensin-aldosterone system inhibitors (RAAS-I) (**A**) and diuretics (**B**) in all patients (n = 311) are shown. The definitions of the indicated medication profiles were described in the *Methods* section. ^*^P < 0.04 by one way analysis of variance, followed by Scheffe’s test for three subjects. NS; not significant.

**Figure 4:**
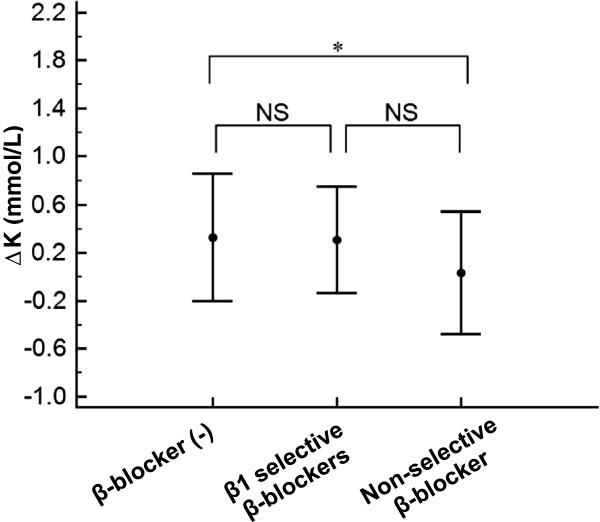
**A comparison of ΔK among the medication profiles of β-blockers on admission.** A comparison of ΔK among the medication profiles of β-blockers(−) indicating the subjects who did not take any β-blockers on admission (n = 262), those with β1 selective β-blockers use (n = 26) and those with non-selective β-blocker use (n = 23) on admission is shown. ^*^P < 0.05 by one way analysis of variance, followed by Scheffe’s test for three subjects. NS; not significant.

To evaluate the involvement of RAAS-I and diuretics, we examined the influence of the changes in each medication profile on ΔK. The group of RAAS-I “newly administered” during hospitalization showed a significantly larger ΔK in comparison to the “no change” group (Figure 3A, P < 0.04). The other variations of medication profiles including RAAS-I discontinuation and diuretics with any changes had no impact on ΔK (Figure 3A and 3B). We next examined the influence of each β-blocker use at the time of admission on ΔK. The subjects with non-selective β-blocker use on admission, but not those with β1 selective β-blockers use, exhibited significantly smaller ΔK values compared to the subjects who did not take any β-blockers on admission (Figure 4, P < 0.05).

To assess the independent determinants of ΔK, a multiple regression analysis was performed (Table 3). The medication profile of RAAS-I and the use of β-blockers on admission no longer had any impact on ΔK, although the profile of non-selective β-blocker use showed a tendency toward negative correlation that did not achieve statistical significance. Intriguingly, the plasma glucose level during ischemic attack (on admission) was found to be the only independent factor positively correlated with ΔK (P = 0.005). In contrast, the independent factors negatively correlated with ΔK were HbA1c (P = 0.04) and K on admission (P < 0.001). Meanwhile, there was a significant positive correlation between plasma glucose level on admission and HbA1c (P < 0.001 in a linear regression analysis, data not shown).

**Table 3:** The results of a multiple regression analysis of ΔK (n = 311)

**Significant variables**	**Regression coefficients**	**Standard error**	**Standard regression coefficients**	**P**
K on admission	−0.77	0.049	−0.659	<0.001
Glucose on admission	0.001	0.0005	0.177	0.005
HbA1c	−0.054	0.026	−0.127	0.04
Use of β-blockers on admission (β1 selective)	0.047	0.079	0.025	0.551
(non-selective)	−0.161	0.084	−0.8	0.058
RAAS-I newly administered	0.05	0.047	0.048	0.286
Discontinued	0.04	0.119	0.014	0.736
Diuretics newly administered	0.1	0.085	0.052	0.24
Discontinued	0.114	0.143	0.032	0.426

No significant variables (other than the medication profiles): BMI, Age, BP, eGFR, LVEF, BNP.

Dependent variable: ΔK.

Explanatory variables: BMI, Glucose, Age, BP, K on admission, HbA1c, eGFR, RASS-I or diuretics newly administered,

RAAS-I or diuretics discontinued, LVEF, BNP, use of β-blocker on admission.

K: potassium.

We investigated the association of ΔK with the disease severity and clinical course to evaluate the clinical implications of ΔK during ischemic attack of ACS. A longer hospitalization period, as well as a higher incidence of MI and higher peak CK level were observed in patients with a larger ΔK (Table 4). It has been reported that a decreased serum K level per se increases cardiovascular risks [9,13,14]. Therefore, we also examined the impact of serum K level at the time of admission on the clinical consequences and found a higher incidence of MI and higher peak CK level in the patients with lower K level (K < 4.1) on admission (Table 5).

**Table 4:** The impact of ΔK on disease severity and clinical course

	**ΔK < 0.3 (n = 136)**	**ΔK≧0.3 (n = 175)**	
Time of hospital stay (days)	10.5 ± 10.8	13.8 ± 8.9	P = 0.0039
Myocardial Infarction	59 (43.4%)	124 (70.9%)	P < 0.001
Peak Creatine Kinase (U/L)	1010.0 ± 1540.3	2004.1 ± 2329.0	P < 0.001

**Table 5:** The impact of K level on admission on disease severity and clinical course

	**K≧4.1 (n = 169)**	**K < 4.1 (n = 142)**	
Time of hospital stay (days)	11.6 ± 9.5	13.2 ± 10.3	NS
Myocardial Infarction	88 (52.1%)	95 (66.9%)	P = 0.011
Peak Creatine Kinase (U/L)	1343.5 ± 1853.7	1838.3 ± 2296.3	P = 0.04

## Discussion

In the present study, we found that in ACS patients, serum K level is significantly decreased during ischemic attack compared to the stable phase in individual subjects. We examined multiple clinical factors affecting the degree of the potassium dip (as indicated by ΔK), and found that the plasma glucose level during ischemic attack was the sole factor which was positively correlated with ΔK. In contrast, HbA1c level was negatively correlated with ΔK.

There was no link between the medication profiles and ΔK, such as the use of ACE-inhibitors, ARBs, MR-inhibitors and diuretics, after adjusting for any potential confounders of ΔK, even in patients who had been on these medications before admission (Table 3). Moreover, the renal function, as indicated by eGFR did not affect the potassium dip, suggesting that the potassium kinetics during ischemic attack are not simply regulated by renal elimination.

Although a couple of potential mechanisms for this K decrease have been proposed [1-4,14], the precise mechanisms remain to be elucidated. Meanwhile, we have previously reported that K level decreases with the severity of heart failure if renal function is preserved [15].

It is possible that insulin stimulates an intracellular K shift into the cardiac and skeletal muscles via Na^+^/K^+^ATPase activation, leading to the decrease in serum K level [16-19]. In the present study, plasma glucose level during attack was positively correlated with HbA1c level, thus, at least theoretically, HbA1c level would also be positively correlated with ΔK. However, we found that HbA1c was not correlated with ΔK in the simple regression analysis and was actually negatively correlated with ΔK after adjusting for any potential confounders. Although this negative correlation is relatively weak, one can still say that an elevated glucose level during attack is tightly associated with an enhanced ΔK, regardless of the severity of diabetic condition. These findings suggest that insulin resistance may have had a role in attenuating the potassium dip, and that there are other serum K lowering systems that may overwhelm the effects of insulin resistance, as discussed below. Further investigations are required to fully demonstrate that the tight correlation between glucose and ΔK overwhelms insulin resistance using other parameters, such as homeostasis model assessment ratio (HOMA-R), which was not available in the current study.

The systemic sympathetic nerve system can be activated by ischemic stress, and elevated catecholamines stimulate Na^+^/K^+^ATPase primarily via β2-adrenergic receptor [1-4,20,21]. In fact, in the present study, non-selective β-blocker use on admission, but not β1 selective β-blocker use, reduced ΔK in the one-way ANOVA (Figure 4), although the multiple regression analyses showed only a tendency toward a negative correlation between non-selective β-blocker use and ΔK that did not achieve statistical significance (Table 3). These data indicate that catecholaminergic effects via β2-adrenergic receptor would be partially involved, but that they cannot explain the entire extent of the potassium dip.

Sodium-proton exchanger (NHE) also stimulates Na^+^/K^+^ATPase [16]. Although insulin is one of the NHE activators [22,23], diabetic condition (namely, hyperinsulinemia) as indicated by increased HbA1c, rather reduces ΔK. It is possible that other factors, such as intracellular acidification and some neurohumoral regulators, including the renin-angiotensin-aldosterone system are directly involved in the activation of NHE under the presence of insulin resistance [24,25].

One can infer that the intracellular components, including potassium, leak out when cardiomyocytes are damaged by ischemic attacks, thus leading to an increase in serum K level during attack (namely, ΔK reduction) just like cardiac enzymes, such as CK(−MB). However, the present study demonstrated that a higher peak CK level was observed in patients with a larger ΔK and a lower K (K < 4.1) on admission (Table 4). Moreover, the subanalysis with MI subjects (n = 188) in a multiple regression analysis demonstrated that ΔK was not significantly correlated with peak CK level (if anything, ΔK was actually found to be positively correlated with peak CK level in a simple regression analysis, data not shown, n = 188, P = 0.002), thus suggesting that ΔK reflects the severity of ischemic stress rather than the extent of cellular injury.

In accord with previous reports demonstrating that a decrease in serum K level during the acute phase of ACS increases the risk of cardiovascular events [1,3-7], we found in the present study that more severe ischemia was observed in patients with a lower K level on admission (Table 5). Moreover, the present study revealed that a lower serum K level on admission was associated with a larger ΔK (Figure 2b, Tables 2 and 3). Considering that the patients who presented with lower K level during ischemic attack did not necessarily continue to exhibit relatively low K concentration during stable phase, these data indicate that lower K subjects are more susceptible to larger potassium dip, thus suggesting that serum K level on admission per se reflects disease severity.

The main limitation of this study was that we did not measure the hormonal changes (i.e. serum concentrations of catecholamine, insulin, aldosterone, ACTH, cortisol etc.) or the urinary potassium concentration during attacks. Therefore, the mechanisms described above still remain speculative. Moreover, it would be very interesting to determine the K and glucose concentrations in the coronary sinus, so that the local potassium kinetics in the ischemic heart, where glucose becomes an important preferential substrate for metabolism [26,27], could be evaluated.

## Conclusions

The present study clearly showed a transient decrease to exist in serum K level during ischemic attack of ACS. The degree of the potassium dip was tightly correlated with glucose level, which overwhelmed the diabetic condition and the variations in the medication profiles. The study suggests that the potassium dip indicates the severity of acute ischemic stress, thus promoting awareness of the significance of monitoring K level in parallel with glucose level in patients with ACS, especially in severe cases.

## Abbreviations

ACS: Acute coronary syndrome; BMI: Body mass index; BNP: B-type natriuretic peptide; CK: Creatine kinase; DM: Diabetes mellitus; eGFR: Estimated glomerular filtration rate; HbA1c: Glycohemoglobin; HOMA-R: Homeostasis model assessment ratio; LVEF: Left ventricular ejection fraction; MI: Myocardial infarction; NHE: Sodium-proton exchanger; RAAS-I: Rennin-angiotensin-aldosterone system inhibitors.

## Competing interests

There are no conflicts of interest to declare.

## Authors’ contributions

HS collected the data, performed the statistical analyses, and wrote the manuscript. TN conceived of the research hypothesis and analyses, wrote and edited the manuscript. KK, MM and DK performed the statistical analyses and edited the manuscript. KO, KM and TO participated in the design and coordination of the study and collected the data. MY conceived of the study, and participated in its coordination and edited the manuscript. All authors read and approved the final manuscript.

## Supplementary Material

Additional file 1 **Medication profile. **ACE-I: angiotensin-converting-enzyme inhibitor, ARB: angiotensin-receptor-blocker, RAAS-I: renin-angiotensin-aldosterone system inhibitors. Click here for file
