# Supplementary material for: Transient decrease in serum potassium level during ischemic attack of acute coronary syndrome: Paradoxical contribution of plasma glucose level and glycohemoglobin
Source: Cardiovasc Diabetol. 2013 Jan 4;12:4. doi: 10.1186/1475-2840-12-4 (PMC3561250; doi:10.1186/1475-2840-12-4)
Supplement: Additional file 1 — Medication profile. ACE-I: angiotensin-converting-enzyme inhibitor, ARB: angiotensin-receptor-blocker, RAAS-I: renin-angiotensin-aldosterone system inhibitors. [file 1475-2840-12-4-S1.doc]

**Additional file 1 Medication profile.**

| Drugs | Number on admission | Mean dose (mg/day) | Number at discharge | Mean dose (mg/day) |
| --- | --- | --- | --- | --- |
| ACE-I | 26 |  | 157 |  |
| Imidapril | 15 | 6.0+2.1 | 75 | 4.9+1.6 |
| Enalapril | 6 | 5.8+2.0 | 54 | 4.8+1.8 |
| Perindopril | 2 | 4.0 | 25 | 3.9+1.8 |
| Lisinopril | 1 | 10 | 1 | 10 |
| Trandolapril | 1 | 1 | 0 |  |
| Temocapril | 1 | 2 | 0 |  |
| Alacepril | 0 |  | 2 | 37.5+17.7 |
| ARB | 98 |  | 97 |  |
| Valsartan | 29 | 86.9+45.4 | 34 | 74.7+41.0 |
| Candesartan | 23 | 6.7+2.4 | 13 | 6.9+2.7 |
| Losartan | 18 | 42.4+19.7 | 14 | 42.0+21.7 |
| Telmisartan | 14 | 23.8+9.6 | 19 | 37.9+13.2 |
| Olmesartan | 13 | 23.8+9.6 | 17 | 22.9+8.5 |
| Irbesartan | 1 | 50 | 0 |  |
| Mineralocorticoid receptor inhibitors | |  |  |  |
| Spironolactone | 5 | 27.0+14.4 | 28 | 24.5+6.5 |
| Eplerenone | 0 |  | 4 | 25 |
| RAAS-I free | 189 |  | 56 |  |
| β-blockers (non-selective) | 49 (23) |  | 131 (110) |  |
| Loop diuretics | 16 |  | 37 |  |
| Thiazide | 8 |  | 4 |  |

ACE-I: angiotensin-converting-enzyme inhibitor, ARB: angiotensin-receptor-blocker, RAAS-I: renin-angiotensin-aldosterone system inhibitors
